# Prioritizing network communities

**DOI:** 10.1038/s41467-018-04948-5

**Published:** 2018-06-29

**Authors:** Marinka Zitnik, Rok Sosič, Jure Leskovec

**Affiliations:** 1 Computer Science Department, Stanford University, 353 Serra Mall, Stanford, CA 94305 USA; 2 Chan Zuckerberg Biohub, 499 Illinois St., San Francisco, CA 94158 USA

## Abstract

Uncovering modular structure in networks is fundamental for systems in biology, physics, and engineering. Community detection identifies candidate modules as hypotheses, which then need to be validated through experiments, such as mutagenesis in a biological laboratory. Only a few communities can typically be validated, and it is thus important to prioritize which communities to select for downstream experimentation. Here we develop CRank, a mathematically principled approach for prioritizing network communities. CRank efficiently evaluates robustness and magnitude of structural features of each community and then combines these features into the community prioritization. CRank can be used with any community detection method. It needs only information provided by the network structure and does not require any additional metadata or labels. However, when available, CRank can incorporate domain-specific information to further boost performance. Experiments on many large networks show that CRank effectively prioritizes communities, yielding a nearly 50-fold improvement in community prioritization.

## Abstract (web summary)

Community detection allows one to decompose a network into its building blocks. While communities can be identified with a variety of methods, their relative importance can’t be easily derived. Here the authors introduce an algorithm to identify modules which are most promising for further analysis.

## Introduction

Networks exhibit modular structure^1^ and uncovering it is fundamental for advancing the understanding of complex systems across sciences^2,3^. Methods for community detection^4^, also called node clustering or graph partitioning, allow for computational detection of modular structure by identifying a division of network’s nodes into groups, also called communities^5–10^. Such communities provide predictions/hypotheses about potential modules of the network, which then need to be experimentally validated and confirmed. However, in large networks, community detection methods typically identify many thousands of communities^6,7^ and only a small fraction can be rigorously tested and validated by follow-up experiments. For example, gene communities detected in a gene interaction network^11^ provide predictions/hypotheses about disease pathways^2,3^, but to confirm these predictions scientists have to test every detected community by performing experiments in a wet laboratory^3,8^. Because experimental validation of detected communities is resource-intensive and generally only a small number of communities can be investigated, one must prioritize the communities in order to choose which ones to investigate experimentally.

In the context of biological networks, several methods for community or cluster analysis have been developed^2,3,12–15^. However, these methods crucially rely and depend on knowledge in external databases, such as Gene Ontology (GO) annotations^16^, protein domain databases, gene expression data, patient clinical profiles, and sequence information, in order to calculate the quality of communities derived from networks. Furthermore, they require this information to be available for all communities. This means that if genes in a given community are not present in a gene knowledge database then it is not possible for existing methods to even consider that community. This issue is exacerbated because knowledge databases are incomplete and biased toward better-studied genes^11^. Furthermore, these methods do not apply in domains at the frontier of science where domain-specific knowledge is scarce or non-existent, such as in the case of cell–cell similarity networks^17^, microbiome networks^18,19^, and chemical interaction networks^20^. Thus, there is a need for a general solution to prioritize communities based on network information only.

Here, we present CRank, a general approach that takes a network and detected communities as its input and produces a ranked list of communities, where high-ranking communities represent promising candidates for downstream experiments. CRank can be applied in conjunction with any community detection method (Supplementary Notes 2 and 5) and needs only the network structure, requiring no domain-specific meta or label information about the network. However, when domain-specific supervised information is available, CRank can integrate this extra information to boost performance (Supplementary Notes 9 and 10). CRank can thus prioritize communities that are well characterized in knowledge bases, such as GO annotations, as well as poorly characterized communities with limited or no annotations. Furthermore, CRank is based on rigorous statistical methods to provide an overall rank for each detected community.

## Results

### Overview of CRank prioritization approach

**Fig. 1:**
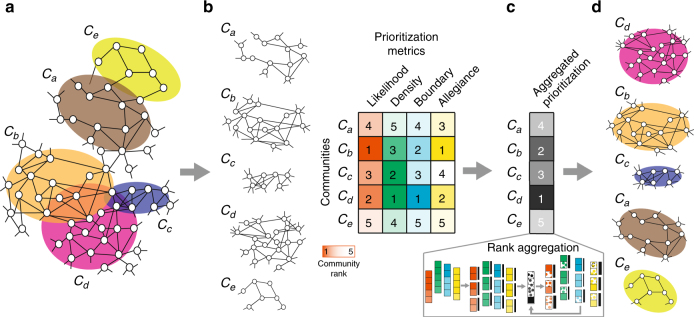
Prioritizing network communities. **a** Community detection methods take as input a network and output a grouping of nodes into communities. Highlighted are five communities, (*C*_*a*_, …, *C*_*e*_), that are detected in the illustrative network. **b** After communities are detected, the goal of community prioritization is to identify communities that are most promising targets for follow-up investigations. Promising targets are communities that are most associated with external network functions, such as cellular functions in protein–protein interaction networks, or cell types in cell–cell similarity networks. CRank is a community prioritization approach that ranks the detected communities using only information captured by the network structure and does not require any external data about the nodes or edges of the network. However, when external information about communities is available, CRank can make advantage of it to further improve performance (Supplementary Notes 9 and 10). CRank starts by evaluating four different structural features of each community: the overall likelihood of the edges in the community (likelihood), internal connectivity (density), external connectivity (boundary), and relationship with the rest of the network (allegiance). CRank can also integrate any number of additional user-defined metrics into the prioritization without any further changes to the method. **c** CRank then applies a rank aggregation method to combine the metrics and **d** produce the final ranking of communities

CRank community prioritization approach consists of the following steps (Fig. 1). First, CRank finds communities using an existing, preferred community detection method (Fig. 1a). It then computes for each community four CRank defined community prioritization metrics, which capture key structural features of the community (Fig. 1b), and then it combines the community metrics via a aggregation method into a single overall score for each community (Fig. 1c). Finally, CRank prioritizes communities by ranking them by their decreasing overall score (Fig. 1d).

CRank uses four different metrics to characterize network connectivity features for each detected community (Methods section). These metrics evaluate the magnitude of structural features as well as their robustness against noise in the network structure. The rationale here is that high-priority communities have high values of metrics and are also stable with respect to network perturbations. If a small change in the network structure—an edge added here, another deleted there—significantly changes the value of a prioritization metric then the community will not be considered high priority. We derive analytical expressions for calculating these metrics, which make CRank computationally efficient and applicable to large networks (Supplementary Note 2). Because individual metrics may have different importance in different networks, a key element of CRank is a rank aggregation method. This method combines the values of the four metrics into a single score for each community, which then determines the community’s rank (see Methods and Supplementary Note 4). CRank’s aggregation method adjusts the impact of each metric on the ranking in a principled manner across different networks and also across different communities within a network, leading to robust rankings and a high-quality prioritization of communities (Supplementary Note 5).

### Synthetic networks

**Fig. 2:**
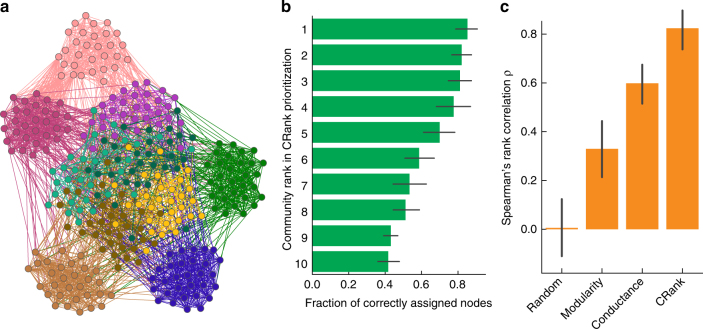
Synthetic networks with planted community structure. **a**–**c** In networks with known modular structure we can evaluate community prioritization by quantifying the correspondence between detected communities and the planted communities. **a** Benchmark networks on *N* = 300 nodes are created using a stochastic block model with 10 planted communities^10^. Each planted community has 30 nodes, which are colored by their planted community assignment. Planted communities use different values for within-community edge probability *p*_in_, five use *p*_in_ = 0.6 and five use *p*_in_ = 0.2. As a result, planted communities with smaller within-community probability *p*_in_ are harder to detect. For each benchmark network we apply a community detection method^6^ to detect communities and then use CRank to prioritize them. CRank produces a ranked list of detected communities. The gold standard rank of each community is determined by how accurately it corresponds to its planted counterpart. **b** Each bar represents one detected community and the bars are ordered by CRank’s ranking with the highest-ranked community located at the top and the lowest ranked community located at the bottom. As a form of validation, the width of each bar corresponds to the fraction of nodes in a community that are correctly classified into a corresponding planted community, with error bars showing the 95% confidence intervals over 500 benchmark networks. A perfect prioritization ranks the bars by decreasing width. Notice that CRank perfectly prioritizes the communities even though it only uses information about the network structure, and has no access to information about the planted communities. **c** Prioritization performance is measured using Spearman’s rank correlation *ρ* between the generated ranking and the gold standard ranking of communities. A larger value of *ρ* indicates a better performance. Across all benchmark networks, CRank achieved average Spearman’s rank correlation of *ρ* = 0.82. Alternative approaches resulted in poorer average performance: ranking based on modularity and conductance achieved *ρ* = 0.33 and *ρ* = 0.60, respectively, whereas random prioritization obtained *ρ* = 0.00

We first demonstrate CRank by applying it to synthetic networks with planted community structure (Fig. 2a). The goal of community prioritization is to identify communities that are most promising candidates for follow-up investigations. Since communities provide predictions about the modular structure of the network, promising candidates are communities that best correspond to the underlying modules. Thus, in this synthetic example, the aim of community prioritization can be seen as to rank communities based on how well they represent the underlying planted communities, while only utilizing information about network structure and without any additional information about the planted communities. We quantify prioritization quality by measuring the agreement between a ranked list of communities produced by CRank and the gold standard ranking (Supplementary Note 7). In the gold standard ranking, communities are ordered in the decreasing order of how accurately each community reconstructs its corresponding planted community.

We experiment with random synthetic networks with planted community structure (Fig. 2a), where we use a generic community detection method^7^ to identify communities and then prioritize them using CRank. We observe that CRank produces correct prioritization—using only the unlabeled network structure, CRank places communities that better correspond to planted communities towards the top of the ranking (Fig. 2b), which indicates that CRank can identify accurately detected communities by using the network structure alone and having no other data about planted community structure. Comparing the performance of CRank to alternative ranking techniques, such as modularity^5^ and conductance^21^ (Supplementary Note 7), we observe that CRank performs 149 and 37% better than modularity and conductance, respectively, in terms of the Spearman’s rank correlation between the generated ranking and the gold standard community ranking (Fig. 2c). Moreover, we observed no correlation with the gold standard ranking when randomly ordering the detected communities. Although zero correlation is expected, poor performance of random ordering is especially illuminating because prioritization of communities is typically ignored in current network community studies.

### Networks of medical drugs with shared target proteins

**Fig. 3:**
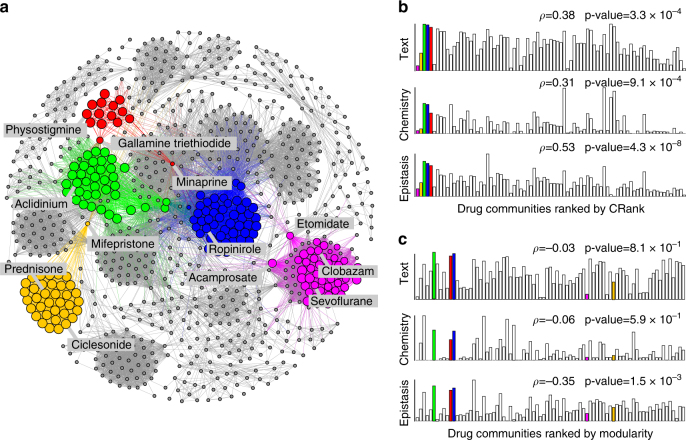
Prioritizing network communities in the network of medical drugs. **a** The network of medical drugs connects two drugs if they share at least one target protein. Communities were detected by a community detection method^7^, and then prioritized by CRank. Highlighted are five highest-ranked communities as determined by CRank. Nodes of the highlighted communities are sized by their score of the Likelihood prioritization metric (Supplementary Note 3). Investigation reveals that these communities contain drugs used to: treat asthma and allergies (e.g., prednisone, ciclesonide; yellow nodes), induce anesthesia or sedation (e.g., clobazam, etomidate, sevoflurane, acamprosate; magenta nodes), block neurotransmitters in central and peripheral nervous systems (e.g., physostigmine, minaprine, gallamine triethiodide; red nodes), block the activity of muscarinic receptors (e.g., acidinium; green nodes), and activate dopamine receptors (e.g., ropinirole; blue nodes). **b**, **c** We evaluate community prioritization against three external chemical databases (Supplementary Note 6) that were not used during community detection or prioritization. For each community we measure: (1) drug-drug interactions between the drugs (“Epistasis”), (2) chemical structure similarity of the drugs (“Chemistry”), and (3) associations between drugs derived from text data (“Text”). We expect that a true high-priority community will have more drug-drug interactions, higher similarity of chemical structure, and stronger textual associations between the drugs it contains. Taking this into consideration, the external chemical databases define three gold standard rankings of communities against which CRank is evaluated. Bars represent communities; bar height denotes similarity of drugs in a community with regard to the gold standard based on external chemical databases. In a perfect prioritization, bars would be ordered such that the heights would decrease from left to right. **b** CRank ranking of drug communities outperforms ranking by modularity **c** across all three chemical databases (as measured by Spearman’s rank correlation *ρ* with the gold standard ranking). CRank ranking achieves *ρ* = 0.38, 0.31, 0.53, while modularity obtains *ρ* = −0.03, −0.06, −0.35

Community rankings obtained by CRank provide a rich source of testable hypotheses. For example, we consider a network of medical drugs where two drugs are connected if they share at least one target protein (Fig. 3a). Because drugs that are used to treat closely related diseases tend to share target proteins^22^, we expect that drugs belonging to the same community in the network will be rich in chemicals with similar therapeutic effects. Identification of these drug communities hence provides an attractive opportunity for finding new uses of drugs as well as for studying drugs’ adverse effects^22^.

After detecting drug communities using a standard community detection method^7^, CRank relies only on the network structure to prioritize the communities. We evaluate ranking performance by comparing it to metadata captured in external chemical databases and not used by the ranking method. We find that CRank assigns higher priority to communities whose drugs are pharmacogenomically more similar (Fig. 3b), indicating that higher-ranked communities contain drugs with more abundant drug-drug interactions, more similar chemical structure, and stronger textual associations. In contrast, ranking communities by modularity score gives a poor correspondence with information in the external chemical databases (Fig. 3c).

We observe that the top-ranked communities are composed from an unusual set of drugs (Fig. 3a and Supplementary Data set), yet drugs with unforeseen community assignment may represent novel candidates for drug repurposing^22^. Examining the highest-ranked communities, we do not expect mifepristone, an abortifacient used in the first months of pregnancy, to appear together with a group of drugs used to treat inflammatory diseases. Another drug with unanticipated community assignment is minaprine, a psychotropic drug that is effective in the treatment of various depressive states^23^. Minaprine is an antidepressant that antagonizes behavioral despair; however, it shares target proteins with several cholinesterase inhibitors. Two examples of such inhibitors are physostigmine, used to treat glaucoma, and galantamine, a drug investigated for the treatment of moderate Alzheimer’s disease^24^. In the case of minaprine, an antidepressant, it was just recently shown that this drug is also a cognitive enhancer that may halt the progression of Alzheimer’s disease^25^. It is thus attractive that CRank identified minaprine as a member of a community of primarily cholinesterase inhibitors, which suggests minaprine’s potential for drug repurposing for Alzheimer’s disease.

The analysis here was restricted to drugs approved for medical use by the U.S. Food and Drug Administration, because these drugs are accompanied by rich metadata that was used for evaluating community prioritization. We find that when CRank integrates drug metadata into its prioritization model, CRank can generate up to 55% better community rankings, even when the amount of additional information about drugs is small (Supplementary Note 10). However, approved medical drugs represent less than one percent of all small molecules with recorded interactions. Many of the remaining 99% of these molecules might be candidates for medical usage or drug repurposing but currently have little or no metadata in the chemical databases. This fact further emphasizes the need for methods such as CRank that can prioritize communities based on network structure alone while not relying on any metadata in external chemical databases.

### Gene and protein interaction networks

CRank can also prioritize communities in molecular biology networks, covering a spectrum of physical, genetic, and regulatory gene interactions^11^. In such networks, community detection is widely used because gene communities tend to correlate with cellular functions and thus provide hypotheses about biological pathways and protein complexes^2,3^.

CRank takes a network and communities detected in that network, and produces a rank-ordered list of communities. As before, while CRank ranks the communities purely based on network structure, the external metadata about molecular functions, cellular components, and biological processes is used to assess the quality of the community ranking (Supplementary Note 7).

Considering highest-ranked gene communities, CRank’s ranking contains on an average five times more communities whose genes are significantly enriched for cellular functions, components, and processes^16^ than random prioritization, and 13% more significantly enriched communities than modularity-based or conductance-based ranking (Supplementary Note 11). For example, in the human protein–protein interaction network, the highest-ranked community by CRank is composed of 20 genes, including *PORCN*, *AQP5*, *FZD6*, *WNT1*, *WNT2*, *WNT3*, and other members of the Wnt signaling protein family^26^ (Supplementary Note 11). Genes in that community form a biologically meaningful group that is functionally enriched in the Wnt signaling pathway processes (*p*-value = 6.4 × 10^−23^), neuron differentiation (*p*-value = 1.6 × 10^−15^), cellular response to retinoic acid (*p*-value = 2.9 × 10^−14^), and in developmental processes (*p*-value = 9.2 × 10^−10^).

Functional annotation of molecular networks is largely unavailable and incomplete, especially when studied objects are not genes but rather other entities, e.g., miRNAs, mutations, single-nucleotide variants, or genomic regions outside protein-coding loci^27^. Thus it is often not possible to simply rank the communities by their functional enrichment scores. In such scenarios, CRank can prioritize communities reliably and accurately using only network structure without necessitating any external databases. Gene communities that rank at the top according to CRank represent predictions that could guide scientists to prioritize resource-intensive laboratory experiments.

### Megascale cell–cell similarity networks

Single-cell RNA sequencing has transformed our understanding of complex cell populations^28^. While many types of questions can be answered using single-cell RNA-sequencing, a central focus is the ability to survey the diversity of cell types and composition of tissues within a sample of cells.

To demonstrate that CRank scales to large networks, we used the single-cell RNA-seq data set containing 1,306,127 embryonic mouse brain cells^29^ for which no cell types are known. The data set was preprocessed using standard procedures to select and filter the cells based on quality-control metrics, normalize and scale the data, detect highly variable genes, and remove unwanted sources of variation^9^. The data set was represented as a weighted graph of nearest neighbor relations (edges) among cells (nodes), where relations indicated cells with similar gene expression patterns calculated using diffusion pseudotime analysis^30^. To partition this graph into highly interconnected communities we apply a community detection method proposed for single-cell data^8^. The method separates the cells into 141 fine-grained communities, the largest containing 18,788 (1.8% of) and the smallest only 203 (0.02% of) cells. After detecting the communities, CRank takes the cell–cell similarity network and the detected communities, and generates a rank-ordered list of communities, assigning a priority to each community. CRank’s prioritization of communities derived from the cell–cell similarity network takes <2 min on a personal computer.

In the cell–cell similarity network, one could assume that top-ranked communities contain highly distinct marker genes^31^, while low-ranked communities contain marker genes whose expression levels are spread out beyond cells in the community. To test this hypothesis, we identify marker genes for each detected community. In particular, for each community we find genes that are differentially expressed in the cells within the community^9^ relative to all cells that are not in the community.

**Fig. 4:**
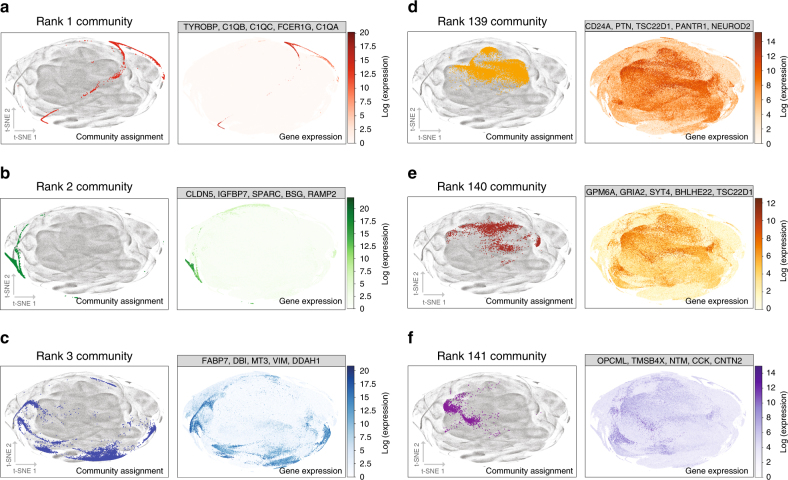
Prioritizing network communities in the megascale cell–cell similarity network. The network of embryonic mouse brain has 1,306,127 nodes representing brain cells^29^. Communities are detected using a community detection method developed for single-cell RNA-seq data^8^ and prioritized using CRank, generating a rank-ordered list of detected communities. **a**–**c** Shown are three communities that are ranked high by CRank; **a** rank 1, **b** rank 2, and **c** rank 3 community. t-SNE projections^39^ show cells assigned to each community. t-SNE is a dimensionality reduction technique that is particularly well suited for visualization of high-dimensional data. Cells assigned to each community are distinguished by color, and all other cells are shown in gray. We investigate the quality of community ranking by examining gene markers for cells in each community^28^. We use the single-cell RNA-seq data set to obtain a gene expression profile for each cell, indicating the activity of genes in the cell. For each community we then identify marker genes, i.e., genes with the strongest differential expression between cells assigned to the community and all other cells^9^. In the t-SNE projection we then color the cells by how active the marker genes are. This investigation reveals that communities ranked high by CRank are represented by clusters of cells whose marker genes have a highly localized expression. For example, marker genes for rank 1 community in **a** (the highest community in CRank ranking) are *TYROBP*, *C1QB*, *C1QC*, *FCER1G*, and *C1QA*. Expression of these genes is concentrated in cells that belong to the rank 1 community. Similarly, marker genes for rank 2 and rank 3 communities are specifically active in cell populations that match well the boundary of each community. **d**–**f** t-SNE projections show cells assigned to 3 low-ranked communities; **d** rank 139, **e** rank 140, and **f** rank 141 community. t-SNE projections are produced using the same differential analysis as in **a**–**c**. Although these communities correspond to clusters of cells in the t-SNE projections, their marker genes have diluted gene expression that is spread out over the entire network, indicating that CRank has correctly considered these communities to be low priority. For example, marker genes for rank 141 community in **f** are *OPCML*, *TMSB4X*, *NYM*, *CCK*, and *CNTN2*, which show a weak expression pattern that is diffused across the entire network

We find that high-ranked communities in CRank contain cells with distinct marker genes, confirming the above hypothesis (average *z*-score of marker genes with respect to the bulk mean gene expression was above 200 and never smaller than 150) (Fig. 4a, b). In contrast, cells in low-ranked communities show a weak expression activity diffused across the entire network and no community-specific expression activity (Fig. 4c, d). Examining cells assigned to the highest-ranked community (rank 1 community) in CRank, we find that most differentially expressed genes are *TYROBP*, *C1QB*, *C1QC*, *FCER1G*, and *C1QA* (at least a 200-fold difference in normalized expression with respect to the bulk mean expression^9^). It is known that these are immuno-regulatory genes and that they play important roles in signal transduction in dendritic cells, osteoclasts, macrophages, and microglia^32^. In contrast, low-ranked communities (Fig. 4 visualizes rank 139, rank 140, and rank 141 communities) contain predominantly cells in which genes show no community-specific expression. Genes in communities ranked lower by CRank hence do not have localized mRNA expression levels, suggesting there are no good marker genes that define those communities^28^. Since the expression levels of mRNA are linked to cellular function and can be used to define cell types^28^, the analysis here points to the potential of using highest-ranked communities in CRank as candidates to characterize cells at the molecular level, even in data sets where no cells are yet classified into cell types.

### Analysis of CRank prioritization approach

The CRank approach can be applied with any community detection method and can operate on directed, undirected, and weighted networks. Furthermore, CRank can also use external domain-specific information to further boost prioritization performance (Supplementary Note 10). Results on diverse biological, information, and technological networks and on different community detection methods show that the second best performing approach changes considerably across networks, while CRank always produces the best result, suggesting that it can effectively harness the network structure for community prioritization (Supplementary Note 8). CRank automatically adjusts weights of the community metrics in the prioritization, resulting in each metric participating with different intensity across different networks (Supplementary Fig. 6). This is in sharp contrast with deterministic approaches, which are negatively impacted by heterogeneity of network structures and network community models employed by different community detection methods. The four CRank community prioritization metrics are essential and complementary. CRank metrics considered together perform on average 45% better than the best single CRank metric, and 26% better than any subset of three CRank metrics (Supplementary Note 8). CRank performs on average 38% better than approaches that combine alternative community metrics (Supplementary Note 8). Furthermore, CRank can easily integrate any number of additional and domain-specific community metrics^2,12–15^, and performs well in the presence of low-signal and noisy metrics (Supplementary Note 9). Furthermore, CRank outperforms alternative approaches that combine the metrics by approximating NP-hard rank aggregation objectives (Supplementary Note 8).

## Discussion

The task of community prioritization is to rank-order communities detected by a community detection method such that communities with best prospects in downstream analysis are ranked towards the top. We demonstrated that prioritizing communities in biological, information, and technological networks is important for maximizing the yield of downstream analyses and experiments. Prior efforts crucially depend on external meta information to calculate the quality of communities with an additional constraint that this information has to be available for all communities. We devised a principled approach for the task of community prioritization. Although the approach does not need any meta information, it can utilize such information if it is available. Furthermore, CRank is applicable even when the meta information is noisy, incomplete, or available only for a subset of communities.

The CRank community ranking is based on the premise that high-priority communities produce high values of community prioritization metrics and that these metrics are stable with respect to small perturbations of the network structure. Our findings support this premise and suggest that both the magnitude of the metrics and the robustness of underlying structural features have an important role in the performance of CRank across a wide range of networks (Supplementary Note 8). CRank can easily be extended using existing network metrics and can also consider new domain-specific scoring metrics (Supplementary Notes 9 and 10). Thus, it would be especially interesting to apply it to networks, where rich meta information exists and interesting domain-specific scoring metrics can be developed, such as protein interaction networks with disease pathway meta information^33^, and molecular networks with genome-wide associations^34^. We believe that the CRank approach opens the door to principled methods for prioritizing communities in large networks and, when coupled with experimental validation, can help us to speed-up scientific discovery process.

## Methods

### Community prioritization model

CRank prioritizes communities based on the robustness and magnitude of multiple structural features of each community. For each feature *f*, we specify a corresponding prioritization metric *r*_*f*_, which captures the magnitude and the robustness of *f*. Robustness of *f* is defined as the change in the value of *f* between the original network and its randomly perturbed version. The intent here is that high-quality communities will have high values of *f* and will also be robust to perturbations of the network structure. We define and discuss specific prioritization metrics later. Here, we first present the overall prioritization model.

Random perturbations of the network are based on rewiring of *α* fraction of the edges in a degree preserving manner^35^ (Supplementary Note 2). Parameter *α* measures perturbation intensity; a value close to zero indicates that the network has only a few edges rewired whereas a value close to one corresponds to a maximally perturbed network, which is a random graph with the same degree distribution as the original network.

Even though the prioritization model is framed conceptually in terms of perturbing the network by rewiring its edges, CRank never actually rewires the network when calculating the prioritization metrics. Network rewiring is a computationally expensive operation. Instead, we derive analytical expressions that evaluate the metrics in a closed form without physically perturbing the network (Supplementary Note 2), which leads to a substantial increase in scalability of CRank.

Given structural feature *f*, we define prioritization metric *r*_*f*_ to quantify the change in the value of *f* between the original and the perturbed network. We want *r*_*f*_ to capture the magnitude of feature *f* in the original network as well as the change in the value of *f* between the network and its perturbed version. We define prioritization metric *r*_*f*_ for community *C* as:

1 $$r_f(C;\alpha ) = \frac{{f(C)}}{{1 + d_f(C,\alpha )}},$$

where *f*(*C*) is the feature value of community *C* in the original network, *α* measures perturbation intensity, *d*_*f*_ (*C*, *α*) = |*f* (*C*) − *f* (*C*|*α*)| is the change of the feature value for community *C* between the network and its *α*-perturbed version, and *f*(*C*|*α*) is the value of feature *f* in the *α*-perturbed version of the network.

Generally, higher priority communities will have higher values of *r*_*f*_. In particular, as *f* can take values between zero and one, then *r*_*f*_ also takes values between zero and one. *r*_*f*_ attains value of zero for community *C* whose value of *f* (*C*) is zero. When *f* (*C*) is nonzero, then *r*_*f*_ (*C*;*α*) down-weights it according to the sensitivity of community *C* to network rewiring. *f* (*C*) is down-weighted by the largest amount when it changes as much as possible under the network perturbation (i.e., *d*_*f*_ (*C*, *α*) = 1). And, *f* (*C*) remains unchanged when community *C* is maximally robust to network perturbation (i.e., *d*_*f*_ (*C*, *α*) = 0).

### Community prioritization metrics

Prioritization metric *r*_*f*_ (*C*) captures the magnitude as well as the robustness of structural feature *f* of community *C*. We define four different community prioritization metrics *r*_*f*_. Through empirical analysis we show that these metrics holistically and non-redundantly quantify different features of network community structure (Supplementary Note 8). Each metric is necessary and contributes positively to the performance of CRank. We combine these metrics into a global ranking of communities using a rank aggregation method that we describe later.

Given a network $G({\cal V},{\cal E},{\cal C})$ with nodes ${\cal V}$, edges ${\cal E}$, and detected communities ${\cal C}$, CRank can be applied in conjunction with any statistical community detection method that allows for computing the following three quantities: (1) the probability of node *u* belonging to a given community *C*, *p*_*C*_(*u*) = *p*(*u* ∈ *C*), (2) the probability of an edge $p(u,v)$ = $p((u,v) \in {\cal E})$, and (3) a contribution of community *C* towards the existence of an edge (*u*,*v*), $p_C(u,v)$ = $p\left( {(u,v) \in {\cal E}|u,v \in C} \right)$. Many commonly used community detection methods allow for computing the above three quantities (Supplementary Note 5).

Our rationale in defining the prioritization metrics is to measure properties that determine a high-quality communϵity, which is also robust and stable with respect to small perturbations of the network. For example, a genuine high-quality community should provide good support for the existence of edges between its members in the original network as well as in the perturbed version. If a small change in the network structure—an edge added here, another deleted there—can completely change the value of the prioritization metric then the community should not be considered high quality. Analogously, a high-quality community should have low confidence for edges pointing outside of the community both in the original as well as in the perturbed network.

### Community likelihood

The community likelihood metric quantifies the overall connectivity of a given community. It measures the likelihood of the network structure induced by the nodes in the community. Note that the metric does not simply count the edges but considers them in a probabilistic way. As such it quantifies how well the observed edges can be explained by the community *C*. The intuition is that high-quality community will contribute a large amount of likelihood to explain the observed edges. We formalize the community likelihood for a given community *C* as follows:

2 $$f_l(C|\alpha ) = \mathop {\prod}\limits_{u \in C} {\kern 1pt} p_C(u)\mathop {\prod}\limits_{v \in C} {\kern 1pt} s_C(u,v|\alpha ),$$

where *s*_*C*_ (*u*, *v*|*α*) is defined as follows:

$$s_C(u,v|\alpha ) = \left\{ {\begin{array}{*{20}{r}} \hfill {p_C(v)p_C(u,v|\alpha )} & \hfill {{\mathrm{if}}\ \left( {u,v} \right) \in {\cal E}}\\ \hfill {p_C(v)(1 - p_C(u,v|\alpha ))} & \hfill {{\mathrm{if}}\ \left( {u,v} \right) \notin {\cal E}.}\end{array}} \right.$$

Here, *p*_*C*_(*u*, *v*|*α*) is a contribution of community *C* towards the creation of edge (*u*, *v*) under network perturbation intensity *α*. We derive analytical expressions for *p*_*C*_(*u*, *v*|*α*) which allows us to compute their values without ever actually perturbing the network (Supplementary Note 2).

Here (and for the other three prioritization metrics) we evaluate the feature in the original network (*f*_*l*_(*C*) = *f*_*l*_(*C*|*α* = 0)) as well as in the slightly perturbed version of the network (*f*_*l*_(*C*|*α* = 0.15)). We then combine the two scores using the prioritization metric formula in Eq. (1).

### Community density

In contrast to community likelihood, which quantifies the contribution of a community to the overall edge likelihood, community density simply measures the overall strength of connections within the community. By considering edge probabilities that are not conditioned on the community *C*, density implicitly takes into consideration potentially hierarchical and overlapping community structures. When a community is nested inside other communities, these enclosing communities contribute to the increased density of community’s internal edges. Formally, we define the density of a community as the joint probability of the edges between community members. Assuming network perturbation intensity *α*, density of community *C* is defined as:

3 $$f_d(C|\alpha ) = \mathop {\prod}\limits_{{(u,v) \in {\cal E}{\kern 1pt} u \in C,v \in C}} {\kern 1pt} p(u,v|\alpha ),$$

where *p*(*u*, *v*|*α*) is the probability of edge (*u*, *v*) under network perturbation intensity *α*. We derive analytical expression for *p*(*u*, *v*|*α*) which allows us to compute their values without ever actually perturbing the network (Supplementary Note 2).

### Community boundary

To complement the internal connectivity measured by community density, community boundary considers the strength of edges leaving the community. A structural feature of a high-quality community is its good separation from the surrounding parts of the network. In other words, a high-quality community should have sharp edge boundary, i.e., $B_C$ = $\{ (u,v) \in {\cal E};u \in C,v \notin C\}$^4^. This intuition is captured by accumulating the likelihood against edges connecting the community with the rest of the network:

4 $$f_b(C|\alpha ) = \mathop {\prod}\limits_{{u \in C{\kern 1pt} v \in {\cal V}\backslash C}} (1 - p(u,v|\alpha )).$$

The evaluation of Eq. (4) takes computational time linear in the size of the network, which is impractical for large networks with many detected communities. To speed up the calculations, we use negative sampling (Supplementary Note 2) to calculate the value of Eq. (4), and thereby reduce the computational complexity of the boundary metric to time that depends linearly on the number of edges leaving the community.

### Community allegiance

Last we introduce community allegiance. We define community allegiance as the preference for nodes to attach to other nodes that belong to the same community. Allegiance measures the fraction of nodes in a community for which the total probability of edges pointing inside the community is larger than probability of edges that point to the outside of the community. For a given community *C* and network perturbation intensity *α*, community allegiance is defined as:

5 $$f_a(C|\alpha ) = \frac{1}{{\left| C \right|}}\mathop {\sum}\limits_{u \in C} {\kern 1pt} \delta \left( {\mathop {\sum}\limits_{v \in N_u \cap C} {\kern 1pt} p(u,v|\alpha ) \ge \mathop {\sum}\limits_{v \in N_u\backslash C} p(u,v|\alpha )} \right),$$

where *N*_*u*_ is a set of network neighbors of *u* and *δ* is the indicator function, *δ*(*x*) = 1 if *x* is true, and *δ*(*x*) = 0, otherwise.

Community has high allegiance if nodes in the community tend to be more strongly connected to other members of the community than to the rest of the network. In a community with no significant allegiance this metric takes a value that is close to zero or changes substantially when the network is only slightly perturbed. However, in the presence of substantial community allegiance, the metric takes large values and is not sensitive to edge perturbation.

### Combining community prioritization metrics

We just defined four community prioritization metrics: likelihood, density, boundary, and allegiance. Each metric on its own provides a useful signal for prioritizing communities (Supplementary Note 8). However, scores of each metric might be biased, have high variance, and behave differently across different networks (Supplementary Fig. 6). It is thus essential to combine the values of individual metrics into a single aggregated score.

**Fig. 5:**
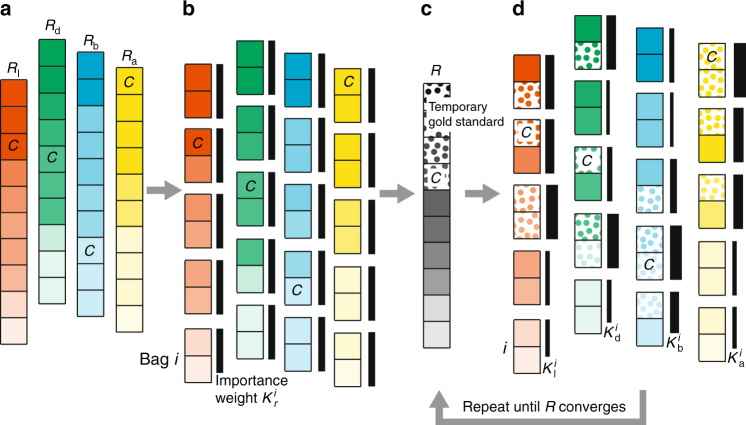
Combining community prioritization metrics without an external gold standard. **a** The rank aggregation algorithm starts with four ranked lists of communities, *R*_*r*_, each one arising from the values of a different community prioritization metric *r* (where *r* is one of “l”— likelihood, “d”—density, “b”—boundary, “a”—allegiance). Communities are ordered by the decreasing value of the metric. We use *C* to indicate the rank of an illustrative community by the community prioritization metrics and at different stages of the algorithm. **b** Each ranked list is partitioned into equally sized groups, called bags. Each bag *i* in ranked list *R*_*r*_ has attached importance weight $K_r^i$ whose initial values are all equal (represented by black bars all of same width). CRank uses the importance weights $K_r^i$ to initialize aggregate prioritization *R* as a weighted average of community ranks *R*_l_, *R*_d_, *R*_b_, *R*_a_. **c** The top-ranked communities (denoted as dotted cells) in the aggregated prioritization *R* serve as a temporary gold standard, which is then used to iteratively update the importance weights $K_r^i$. **d** In each iteration, CRank updates importance weights using the Bayes factor calculation^36^ (Supplementary Note 4). Given bag *i* and ranked list *R*_*r*_, CRank updates importance weight $K_r^i$, based on how many communities from the temporary gold standard appear in bag *i*. Updated importance weights then revise the aggregated prioritization in which the new rank *R*(*C*) of community *C* is expressed as: *R*(*C*) = $\mathop {\sum}\nolimits_r {\kern 1pt} {\mathrm{log}}{\kern 1pt} K_r^{i_r(C)}R_r(C)$, where $K_r^{i_r(C)}$ indicates the importance weight of bag *i*_*r*_(*C*) of community *C* for metric *r*, and *R*_*r*_(*C*) is the rank of *C* according to *r*. By using an iterative approach, CRank allows for the importance of a metric not to be predetermined and to vary across communities

We develop an iterative unsupervised rank aggregation method that, without requiring an external gold standard, combines the prioritization metrics into a single aggregated prioritization of communities. The method is outlined in Fig. 5. It naturally takes into consideration the fact that importance of individual prioritization metrics varies across networks and across community detection methods. The aggregation method starts by representing the values of each prioritization metric with a ranked list. In each ranked list, communities are ordered by the decreasing value of the metric. The method then determines the contribution of each ranked list to the aggregate prioritization by calculating importance weights. The calculation is based on Bayes factors^36–38^, an established tool in statistics. Each ranked list has associated a set of importance weights. Importance weights can vary with rank in the list. The method then calculates the aggregated prioritization of communities in an iterative manner by taking into account uncertainty that is present across different ranked lists and within each ranked list.

To calculate the weights without requiring gold standard, the method uses a two-stage iterative procedure. After initializing the aggregated prioritization, the method alternates between the following two stages until no changes in the aggregated prioritization are observed: (1) use the aggregated prioritization to calculate the importance weights for each ranked list, and (2) re-aggregate the ranked lists based on the importance weights calculated in the previous stage.

The model for aggregating community prioritization metrics, the algorithm, and the analysis of its computational time complexity are detailed in Supplementary Notes 4 and 5 (Supplementary Note 1). The complete algorithm of CRank approach is provided in Supplementary Note 5.

###  Data availability

All relevant data are public and available from the authors of original publications. The project website is at: http://snap.stanford.edu/crank. The website contains preprocessed data used in the paper and additional examples of CRank’s use.

## Electronic supplementary material

Supplementary Information

Description of Additional Supplementary Files

Supplementary Data 1
