# Supplementary material for: Prioritizing network communities
Source: Nat Commun. 2018 Jun 29;9:2544. doi: 10.1038/s41467-018-04948-5 (PMC6026212; doi:10.1038/s41467-018-04948-5)
Supplement: Supplementary file 2 — Description of Additional Supplementary Files [file 41467_2018_4948_MOESM2_ESM.pdf]

Description of Additional Supplementary Files

File Name: Supplementary Data 1
Description: Supplementary Data 1 with prioritization results for the medical drug network
